# A Text-Book of Minor Surgery

**Published:** 1908-03

**Authors:** 


### Books and Magazines.

A TEXT-BOOK OF MINOR SURGERY. By Edward Milton Foote, A. M., M. D., Instructor in Surgery, College of Physicians and Surgeons, Columbia University; Lecturer on Surgery, N. Y. Polyclinic Medical Schools; Visiting Surgeon, New York City Hospital; Visiting Surgeon, St. Joseph's Hospital; Consulting Surgeon, Randall's Island Hospital and Schools; formerly Chief in Surgery at the Vanderbilt Clinic. Illustrated by four hundred and seven engravings, from original drawings and photographs. D. Appleton & Co., New York and London, 1908. Cloth. 713 pages. $5.50, net.

"This book is dedicated to the man at the point of the knife for his grit and patience, and especially for his willingness to be photographed, that others may profit by his misfortune," says the author. Rather a unique dedication. It goes without saying that it is written for the instruction and benefit of the man who essays to hold the other end of the knife. It is altogether a unique book; a radical and very sensible and commendable departure from the stereotyped "Manual of Minor Surgery." Works on minor surgery are usually unsatisfactory compilations or summaries of something that has previously been compiled from something else, and are about as interesting and instructive as a last year's almanac. But this is not a "manual." It is elevated to the dignity of a text-book, and the importance of the subject demands it. There are a good many doctors who are not properly instructed in the essential elements of minor surgery. The mistake that compilers make is in supposing that they are. Dr. Foote probably remembers the time when he was not, and he set to work to learn that which he could not find in the "manuals." And he worked it out at the bedside. In this splendid work he gives us—all of us who are willing to learn—the result of his extended and varied experience. He makes clear the most minute detail,—for every detail is important; tells just how is the best way to do a number of small (and large) things, which some leave to the nurse. No man's education is or can be complete that is not based on a foundation of elementary knowledge. I know a number of physicians who stand well, and pass for fairly good practitioners, who can not write a page of English correctly; some, indeed, who can not even spell correctly. But this book is not intended for the half-educated. The wisest and most experienced may read it with profit, and learn many things which he thought he knew. The style is pleasing and forcible, and I recommend it to my readers as well worth careful study. It is issued in Appleton's usual artistic style. D.

